# QT Prolongation: Psychotropic medication versus illicit drugs

**DOI:** 10.1192/j.eurpsy.2022.1133

**Published:** 2022-09-01

**Authors:** A. Florian, C. Florian, A. Ignat, C. Voinea, L. Popescu, G. Ganea, C. Gherghe, L. Mateescu

**Affiliations:** 1 “Prof. Dr. Al. Obregia” Psychiatry Clinical Hospital, Child And Adolescent Psychiatry, Bucharest, Romania; 2 Emergency Clinical Hospital for Children “M.S.Curie”, Pediatric Cardiology, Bucharest, Romania; 3 Central Military Emergency Hospital “Dr. Carol Davila”, Psychiatry, Bucharest, Romania; 4 Carol Davila University of Medicine and Pharmacy, Child And Adolescent Psychiatry, Bucharest, Romania

**Keywords:** substance abuse, Psychotropic agents, electrocardiogram, QT prolongation

## Abstract

**Introduction:**

Countless substances used for their psychotropic effects may induce adverse cardiac effects, such as QT prolongation. This category of substances holds illicit drugs as well as medications, with their effects influenced by dosage, concomitant use and patient specific factors. The appraisal of cardiac consequences is essential as delayed repolarization may lead to the rare but potentially deadly polymorphic ventricular tachycardia.

**Objectives:**

The goal of this presentation is to underscore the cardiac risks associated with both medication use and substance abuse in order to ensure the suitable psychopharmacological treatment, especially in particular situations of drug using patients.

**Methods:**

The subject of the presentation is a 17-year-old female adolescent hospitalized in our clinic, with multiple substance abuse, as seen in qualitative multidrug test (cannabis, amphetamines, ecstasy, barbiturates, benzodiazepines), previously under complex treatment prescribed by an adult psychiatrist (3 atypical antipsychotics, 1 selective serotonin reuptake inhibitor, 1 anticonvulsant, 1 benzodiazepine). Specialty literature has been reviewed concerning the cardiac effects of both the abuse substances and the psychiatric medications.

**Results:**

Multiple drugs involved may cause a myocardial repolarization delay, the patient having a QTc of 508 msec at the admission. Consequent to parenteral fluids and treatment managing, ECG revealed a decrease to 379 msec 7 days later in the stay. This finding could not be viewed solely as caused by drug use, psychiatric medication or individual factors, but rather as their aggregation.

**Conclusions:**

Psychotropic substances use may lead to QT prolongation, which calls for close cardiac supervision whenever patient’s behaviour warrants or when pharmacologic intervention is required.

**Disclosure:**

No significant relationships.

